# Use of Nab-Paclitaxel Plus Gemcitabine Followed by Hypofractionated Tomotherapy With Simultaneous Integrated Boost in Patients With Locally Advanced Pancreatic Cancer

**DOI:** 10.3389/fonc.2022.782730

**Published:** 2022-03-01

**Authors:** Zhan Shi, Ju Yang, Weiwei Kong, Xin Qiu, Changchang Lu, Juan Liu, Baorui Liu, Juan Du

**Affiliations:** ^1^ The Comprehensive Cancer Centre of Drum Tower Hospital, Medical School of Nanjing University, Clinical Cancer Institute of Nanjing University, Nanjing, China; ^2^ The Comprehensive Cancer Center of Drum Tower Hospital, Clinical College of Traditional Chinese and Western Medicine, Nanjing University of Chinese Medicine, Nanjing, China

**Keywords:** locally advanced pancreatic cancer, nab-paclitaxel, gemcitabine, hypofractionated tomotherapy, simultaneous integrated boost (SIB)

## Abstract

**Background and Purpose:**

A phase 2 study LAPACT indicated nab-paclitaxel plus gemcitabine (AG) improved outcomes of patients with locally advanced pancreatic cancer (LAPC). Conventional radiotherapy failed to show benefit, indicating high dose to volume with high risk of recurrence is needed. The high dose can be delivered through hypofractionated tomotherapy with simultaneous integrated boost (SIB). However, there is a lack of such prospective trials and more data are needed to validate the role of AG plus hypofractionated tomotherapy with SIB in patients with LAPC.

**Materials and Methods:**

Patients with LAPC receiving AG plus tomotherapy at the Nanjing Drum Tower Hospital between 2018 and 2021 were retrospectively analyzed. The treatment was scheduled as follows: nab-paclitaxel 125 mg/m^2^ plus gemcitabine 1,000 mg/m^2^ on days 1 and 8 every three weeks for at least two cycles, followed by hypofractionated tomotherapy with SIB (high dose field: 50 Gy/10 fractions, the remainder: 30 Gy/10 fractions). Then patients were given AG until intolerance or disease progression.

**Results:**

Overall, 22 patients completing the chemoradiotherapy were included. The median follow-up was 15.2 months. After the chemoradiotherapy, 5 patients achieved a partial response (PR), 15 had a stable disease (SD), and another 2 patients were with progressive disease (PD). The median progression-free survival (PFS) and overall survival (OS) were 12.8 months (95% confidence interval [CI] 4.3–21.3 months) and 16.3 months (95% CI 10.9–21.6 months), respectively. The optimal carbohydrate antigen (CA) 19-9 response and chemotherapy cycles ≥6 were correlated with favorable PFS and OS. The most common recurrent pattern was peritoneal dissemination (22.7%) and the locoregional recurrence rate was relatively low (4.5%). Treatments were well-tolerated. The most common grade ≥3 adverse event was thrombocytopenia (13.6%).

**Conclusion:**

This study demonstrated the feasibility of AG followed by hypofractionated tomotherapy with SIB in patients with LAPC. The hypofractionated tomotherapy with SIB was safe and showed high local control rate. Further study with a larger population to validate our data is underway.

## Introduction

Pancreatic cancer is the seventh leading cause of cancer death worldwide, with an estimation of 466,003 deaths and 495,733 new cases worldwide in 2020 according to the GLOBOCAN 2020 (1). In China, pancreatic cancer ranks eighth in cancer-related incidence and sixth in mortality (2). Non-metastatic pancreatic cancer can be classified into 3 groups: resectable, borderline resectable and locally advanced pancreatic cancer (LAPC) (3). Surgery is regarded as the only chance to cure cancer (4). Unfortunately, only 10–20% of patients are eligible for surgery, and approximately 30% are diagnosed as LAPC, with the extensive vascular invasion that precludes curative intended resection (5, 6).

The deaths of patients with LAPC are mainly caused by metastatic spread and uncontrolled local growth (7). Approximately 30–50% of such patients will develop distant metastasis within 3 months (8). Two common regimens, namely, FOLFIRINOX (folinic acid, 5-fluorouracil, irinotecan, and oxaliplatin) and AG (nab-paclitaxel plus gemcitabine), which were initially applied for patients with metastatic disease, have also been evaluated for patients with LAPC. There are a few phase 2 studies that have verified that the AG regimen is active and well-tolerated for patients with LAPC (9–12). A phase 3 trial (NEOPAN) investigating FOLFIRINOX in patients with LAPC is currently ongoing. In addition to the better distant disease control contributed by systemic chemotherapy, better local tumor control is even more critical due to the high morbidity and mortality caused by local failure. Effective chemotherapy regimen coupled with radiotherapy is a strategy to improve the prognosis of patients with LAPC.

However, currently, there is no consensus on the optimal radiotherapy fractionation schedule and the total dose. Previous studies indicated conventional radiotherapy failed to improve survival and the standard dose was insufficient for local tumor control (13). Data regarding the benefits of hypofractionated radiotherapy and dose escalation achieved by simultaneous integrated boost (SIB) are emerging. In terms of hypofractionated radiotherapy, a retrospective study revealed that total dose ≥40 Gy was associated with prolonged overall survival (OS) and progression-free survival (PFS) in patients with unresectable pancreatic adenocarcinoma (14). A phase 2 trial evaluated the hypofractionated radiotherapy of 25 Gy in a single fraction in patients with LAPC and the 1-year local control rate was 94% (15). With intensity-modulated radiation therapy (IMRT)-SIB technique, a set dose is delivered to the total tumor while a higher dose to a volume sparing bowel structures during the same fraction (16). This design is especially suitable for patients with LAPC, because higher dose is needed for tumor-vessel interface (TVI), which is at high risk of recurrence, and lower dose is allowed, which is safe for duodenum and small bowel and effective for tumor areas abutting bowel structures (16). The treatment planning system of helical tomotherapy has advantages in dose coverage, conformity and homogeneity (17), which is ideal for the delivery of hypofractionated radiotherapy with SIB.

Though the combination of an effective regimen such as AG and dose-escalated radiotherapy did show a survival benefit for patients with LAPC, there is a lack of prospective trials. In a phase 2 trial (LAPACT) (9), about 38% of patients with LAPC received radiotherapy after the induction chemotherapy of AG regimen. However, radiotherapy was not part of the protocol of LAPACT; thus, neither details of radiotherapy delivery nor the outcomes of patients receiving chemoradiotherapy were revealed. Therefore, more data are needed to validate the role of the combination of AG regimen and dose-escalated IMRT-SIB in patients with LAPC.

To provide more evidence, we retrospectively analyzed patients with LAPC treated with AG chemotherapy followed by hypofractionated tomotherapy with SIB in our institution. The treatment efficacy and outcomes of patients, namely, OS, PFS, and recurrence pattern were summarized in our study. Furthermore, we investigated the potential associations between clinical characteristics and outcomes of patients.

## Methods and Materials

### Patient Eligibility

Our study was performed in accordance with the guidelines approved by the Ethics Committee of Nanjing Drum Tower Hospital. Written consents of all the enrolled patients were obtained before the treatment. The eligibility criteria included: pathologically confirmed pancreatic adenocarcinoma (ultrasound-guided fine-needle aspiration or resected tumor tissues); unresectable LAPC evaluated by radiologists and surgeons; receiving treatment of AG followed by hypofractionated tomotherapy with SIB (high dose field: 50 Gy/10 fractions, the remainder: 30 Gy/10 fractions) between May 2018 and March 2021. Exclusion criteria were: prior radiotherapy or chemotherapy; stage IV disease; resectable; recurrence disease after resection.

### Chemotherapy

The chemotherapy was given as follows: Nab-paclitaxel 125 mg/m^2^ on days 1 and 8, gemcitabine 1,000 mg/m^2^ on days 1 and 8 every three weeks (AG regimen). Dose modifications were evaluated by the discretion of the oncologists according to the status of patients. Patients received treatment until disease progression, unacceptable toxicities, or refusal of patients.

### Helical Tomotherapy Protocol

Patients were simulated under the following conditions: four hours nothing by mouth, supine position with arms up, immobilized with thermoplastic mask and abdominal compressor. Simulation computed tomography (CT) included scans without or with contrast. An integrated gross tumor volume (iGTV) was defined by the complete extent of tumor delineated on each CT phase. The tumor–vessel interface (TVI) was contoured and included all the portions of vessels with direct contact to tumor. We created a collective planning organ at risk volume (PRV) as a 5 mm expansion from all bowel structures (GI PRV). PTV-Low encompassed a 5 mm expansion of iGTV and TVI without regard to normal structures (PTV-Low: [iGTV + TVI] + 5 mm, 30 Gy/10 fractions). We generated PTV-High from iGTV and TVI while subtracting the GI PRV (PTV-High: [iGTV + TVI] − GI PRV, 50 Gy/10 fractions). The clinical objective was to cover >98% of PTV-Low with 30 Gy and ≥90–95% of PTV-High with 50 Gy. Normal tissue constrains were described in previous studies (16, 18).

### Response Evaluation and Follow Up

Treatment responses were evaluated every 2–3 months by CT and/or magnetic resonance imaging (MRI) according to response evaluation criteria in solid tumors (RECIST version 1.1) (19). CT scans were performed within 4–6 weeks after the end of radiotherapy to estimate the treatment response of radiotherapy. Primary outcomes included the tumor response, OS, and PFS. Optimal carbohydrate antigen (CA) 19-9 response was defined as normalization and/or ≥50% decline compared with baseline. The treatment-related toxicities were assessed according to the common toxicity criteria for adverse events (CTCAE) version 4.03.

### Statistical Analyses

All the statistical analyses were performed using IBM SPSS Version 20. Kaplan–Meier curves were plotted to assess the cumulative probability of observed outcomes. Potential associations between patient- and treatment-related factors and outcomes were assessed with a Cox proportional hazards model, with consideration of time to the event. Two-sided *P <*0.05 was considered statistically significant. Factors with *P <*0.05 in the univariate analysis were entered into the multivariate analysis.

## Results

### Patients’ Characteristics

As shown in **Supplementary Figure 1**, between May 2018 and March 2021, there were 76 patients with pancreatic cancer receiving hypofractionated tomotherapy in our institution. The number of patients with distant metastasis, LAPC at diagnosis and recurrence after surgery were 16, 34, and 26, respectively. In those patients with LAPC, 24 patients received AG chemotherapy, and 2 patients received radiotherapy with a total dose <50 Gy. Finally, 22 patients completed hypofractionated tomotherapy with SIB (high dose field: 50 Gy/10 fractions, the remainder: 30 Gy/10 fractions) and were analyzed in our study. As shown in **Table 1**, the median age at diagnosis was 63 years old (range 41–78 years), 27% of the included patients were male and 50% of tumors were located at the head and neck of the pancreas. Tumor over 4 cm in diameter was observed in 8 patients, and the CA19-9 levels were increased in 16 patients at diagnosis. Four patients presented with diabetes.

**Table 1:** Characteristics of patients with LAPC.

Characteristics	Cases	%
Age, years		
<65	13	59
≥65	9	41
Sex		
Male	6	27
Female	16	73
BMI, kg/m^2^		
<24	18	82
≥24	4	18
ECOG PS		
0	4	18
1	18	82
Smoking history		
Yes	8	36
No	14	64
Alcohol		
Yes	6	27
No	16	73
Diabetes		
Yes	4	18
No	18	82
Hypertension		
Yes	5	23
No	17	77
Family history of cancer		
Yes	3	14
No	19	86
Tumor location		
Head & Neck	11	50
Body & Tail	11	50
Tumor size, cm		
<4	14	64
≥4	8	36
CA19-9, U/ml		
<27	6	27
≥27	16	73
Chemotherapy cycles		
<6	7	32
≥6	15	68

LAPC, locally advanced pancreatic cancer; BMI, body mass index; ECOG PS, Eastern Cooperative Oncology Group Performance Status; CA 19-9, carbohydrate antigen 19-9.

### Primary Outcomes: OS, PFS, and Treatment Responses

**Figure 1:**
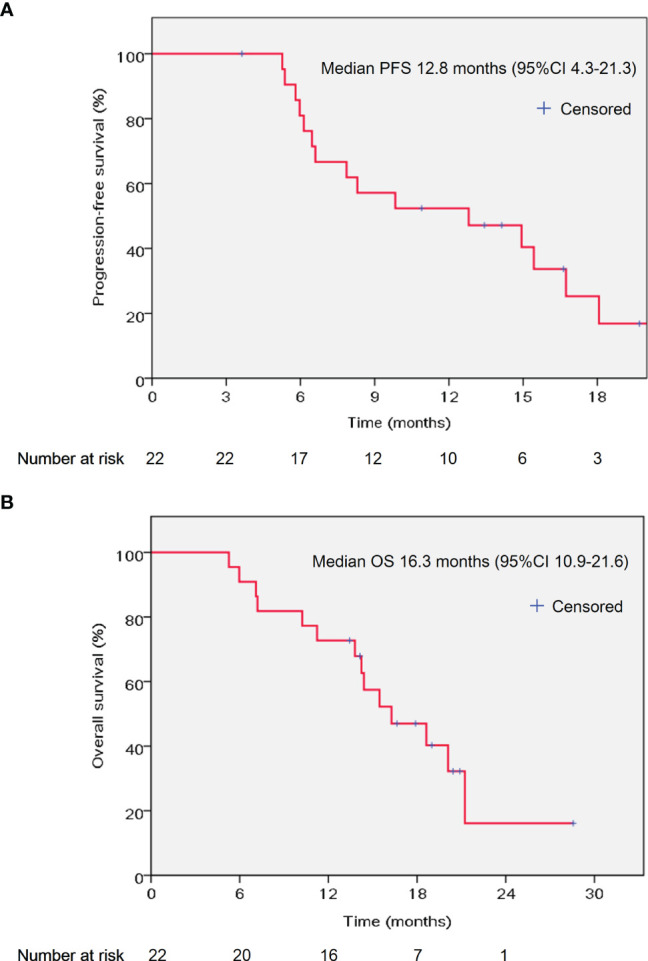
Kaplan–Meier curves of PFS **(A)** and OS **(B)** in the studied population. PFS, progression-free survival; OS, overall survival; CI: confidence interval.

**Figure 2:**
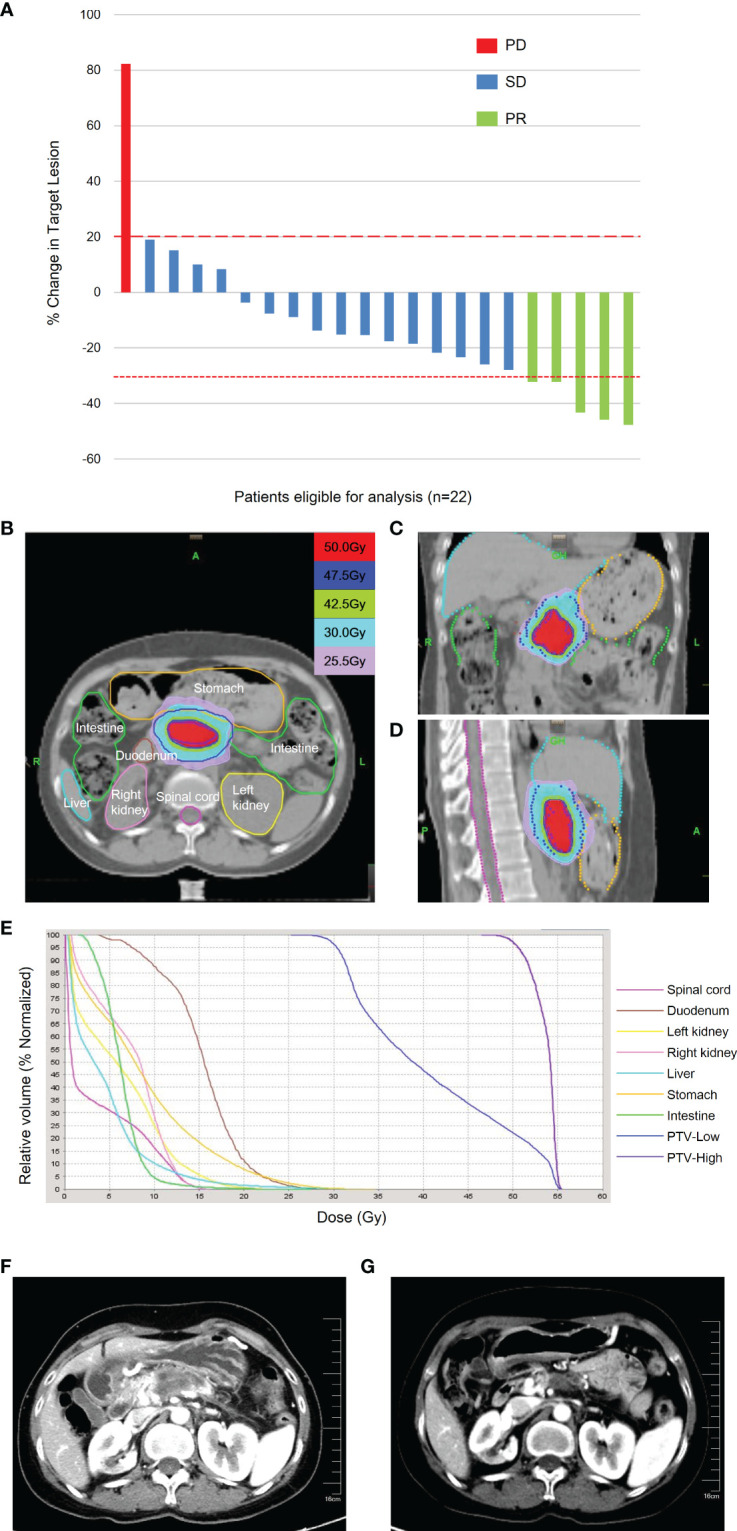
Best treatment responses and representative radiotherapy plan. **(A)** Best percentage change in tumor size from baseline during treatment. The dashed lines above and below the x-axis represented 20% increase and 30% decrease of target lesions from baseline, respectively. Different color codes represented different tumor response (red, PD; blue, SD; and green, PR). The isodose lines of axial plane **(B)**, coronal plane **(C)**, and sagittal plan **(D)**. Dose-volume histogram **(E)** was shown. The different doses were represented with different colors. **(F)** Enhanced CT demonstrated invasion of vessels by tumor before chemoradiotherapy. **(G)** Enhanced CT revealed the volume of tumors with vessel invasion decreased after chemoradiotherapy. PD, progressive disease; SD, stable disease; PR, partial response; LAPC, locally advanced pancreatic cancer; CT, computed tomography.

At the time of writing the manuscript, the median follow up was 15.2 months (range 5.3–28.6 months). All patients received AG chemotherapy for at least two cycles. The median chemotherapy cycle was 6 cycles (range 2–22 cycles). The median PFS was 12.8 months (95% CI 4.3–21.3 months), and the median OS was 16.3 months (95% CI 10.9–21.6 months) (**Figure 1**). The percentage change of tumor dimension and best responses to treatment during the treatment are described in **Figure 2A** and **Supplementary Table 1**. Overall, five patients (22.7%) achieved a radiographic partial response (PR), while 15 (68.2%) were with stable disease (SD) and two patients (9.1%) with progressive disease (PD) (one patient had new tumor lesions) according to RECIST version 1.1. The representative hypofractionated tomotherapy with SIB plan and CT images of a patient before and after the chemoradiotherapy were displayed in **Figures 2B–G**.

### Recurrence Pattern

At the latest follow-up, 3 patients remained progression-free, 3 patients were lost to follow up and relapse occurred in another 16 patients. As shown in **Table 2**, among the 16 patients who experienced treatment failure, local progression occurred in 1 patient (4.5%), and 15 patients developed distant metastases as their first site of treatment failure (68.2%).

**Table 2:** Pattern of recurrence.

Recurrence pattern	Cases	%
Locoregional recurrence	1	4.5
Distant recurrence	15	68.2
Peritoneal dissemination	5	22.7
Liver	4	18.2
Lung	2	9.1
Bone	2	9.1
Multiple sites	2	9.1

### Optimal CA19-9 Response and More Chemotherapy Cycles Were Associated With Favorable Outcomes

**Figure 3:**
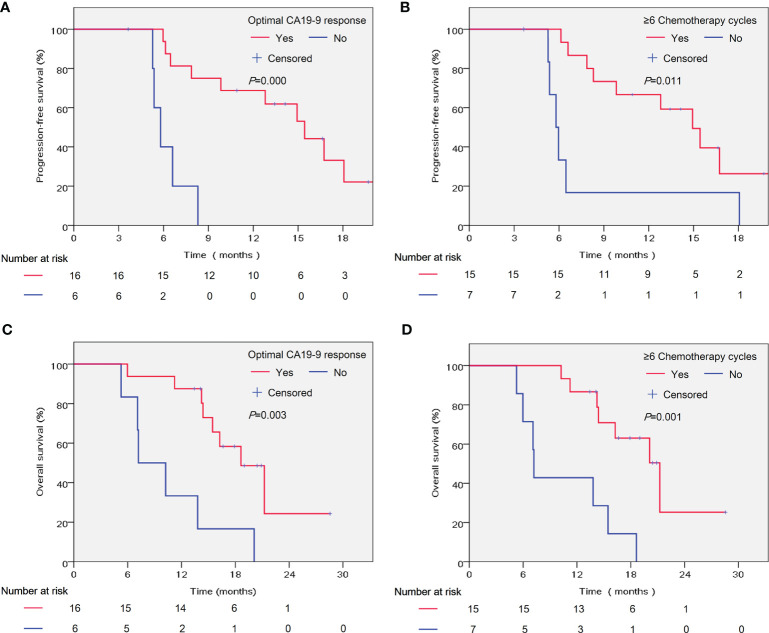
Kaplan–Meier analysis of patient significant factors in the multivariate analyses and outcomes of patients. PFS was analyzed according to the optimal CA19-9 response **(A)** and chemotherapy cycles **(B)**. OS was analyzed according to the optimal CA19-9 response **(C)** and chemotherapy cycles **(D)**. PFS, progression-free survival; OS, overall survival. Optimal CA19-9 response: Normalization and/or ≥50% decline compared with baseline.

Furthermore, we investigated the potential associations between patient- and treatment-related factors and outcomes. In the univariate analysis, we found that optimal CA19-9 response (hazard ratio [HR] = 0.117, 95% confidence interval [CI] 0.031–0.450, *P* = 0.002) and more chemotherapy cycles (HR = 0.267, 95% CI 0.091–0.786, *P* = 0.016) were significantly correlated with prolonged PFS (**Table 3**). In the multivariate analysis, the correlation with favorable PFS was still statistically significant (optimal CA19-9 response, HR = 0.106, 95% CI 0.026–0.436, *P* = 0.002; chemotherapy cycles ≥6, HR = 0.242, 95% CI 0.075–0.783, *P* = 0.018) (**Table 3**). As shown in **Table 3**, optimal CA19-9 response and chemotherapy cycles ≥6 were significantly associated with improved OS in both the univariate analysis (optimal CA199 response, HR = 0.207, 95% CI 0.067–0.636, *P* = 0.006; chemotherapy cycles ≥6, HR = 0.165, 95% CI 0.051–0.530, *P* = 0.002) and multivariate analysis (optimal CA19-9 response, HR = 0.253, 95% CI 0.075–0.856, *P* = 0.027; chemotherapy cycles ≥6, HR = 0.194, 95% CI 0.057–0.661, *P* = 0.009), which was similar to the results for PFS. The significant association between optimal CA19-9 response and chemotherapy cycles ≥6 and outcomes of patients, namely, PFS and OS was similarly indicated in the Kaplan–Meier analyses (**Figure 3**).

**Table 3:** Stepwise univariate and multivariate Cox regression analyses to identify predictors of PFS and OS.

Characteristics		Univariate analysis of PFS			Multivariate analysis of PFS			Univariate analysis of OS			Multivariate analysis of OS		
		HR	95%CI	*P*	HR	95%CI	*P*	HR	95%CI	*P*	HR	95%CI	*P*
Age, years	<65	1(Ref)		0.571			NA	1(Ref)		0.842			NA
	≥65	1.356	0.472-3.899					0.898	0.309-2.604				
Sex	Male	1(Ref)		0.171			NA	1(Ref)		0.183			NA
	Female	0.430	0.128-1.441					0.465	0.151-1.434				
BMI, kg/m^2^	<24	1(Ref)		0.755			NA	1(Ref)		0.684			NA
	≥24	1.227	0.340-4.426					1.308	0.359-4.765				
ECOG PS	0	1(Ref)		0.662			NA	1(Ref)		0.635			NA
	1	1.409	0.303-6.552					0.680	0.139-3.341				
Smoking history	No	1(Ref)		0.306			NA	1(Ref)		0.384			NA
	Yes	1.710	0.612-4.781					0.595	0.184-1.918				
Alcohol	No	1(Ref)		0.688			NA	1(Ref)		0.442			NA
	Yes	0.788	0.246-2.526					0.603	0.166-2.188				
Diabetes	No	1(Ref)		0.406			NA	1(Ref)		0.608			NA
	Yes	0.573	0.154-2.130					1.363	0.418-4.442				
Hypertension	No	1(Ref)		0.923				1(Ref)		0.174			
	Yes	1.058	0.334-3.350					2.229	0.702-7.075				
Family history of cancer	No	1(Ref)		0.504			NA	1(Ref)		0.621			NA
	Yes	1.555	0.426-5.675					1.387	0.379-5.076				
Tumor location	Head& Neck	1(Ref)		0.409			NA	1(Ref)		0.260			NA
	Body& Tail	0.651	0.235-1.804					0.525	0.171-1.611				
Tumor size, cm	<4	1(Ref)		0.253			NA	1(Ref)		0.339			NA
	≥4	1.961	0.618-6.227					1.710	0.569-5.134				
Optimal CA19-9 response	No	1(Ref)		**0.002 ^a^**	1(Ref)		**0.002 ^b^**	1(Ref)		**0.006 ^a^**			**0.027 ^b^**
	Yes	0.117	0.031-0.450		0.106	0.026-0.436		0.207	0.067-0.636		0.253	0.075-0.856	
Chemotherapy cycles	<6			**0.016 ^a^**			**0.018 ^b^**	1(Ref)		**0.002 ^a^**			**0.009 ^b^**
	≥6	0.267	0.091-0.786		0.242	0.075-0.783		0.165	0.051-0.530		0.194	0.057-0.661	

Abbreviations：PFS, progression-free survival；OS, overall survival; BMI, body mass index; ECOG PS, eastern cooperative oncology group performance status; CA 19-9, carbohydrate antigen 19-9; HR, hazard ratio; Ref, reference; CI, confidence interval; NA, not applicable; Optimal CA19-9 response: Normalization and/or ≥50% decline compared with baseline.

a: Two-sided P<0.05 was considered statistically significant.

b: Factors with P<0.05 in the univariate analysis were entered into the multivariate analysis.

The bold values represented P<0.05.

### Treatment-Related Toxicities

Overall, no duodenal bleeding occurred after chemoradiotherapy in our study. The most common hematological toxicity was grade 1/2 anemia, and the main severe toxicity grade ≥3 was thrombocytopenia. In addition, 8 of 22 patients had fatigue during the treatment. The vomiting and nausea are common gastrointestinal toxicities. The adverse events are summarized in **Supplementary Table 2**. All toxicities were relieved after supportive treatment. No treatment-related death occurred.

## Discussion

In the current study, we demonstrated the feasibility of AG chemotherapy and hypofractionated tomotherapy with SIB in patients with LAPC. Of note, high local control rate and relatively long PFS were achieved in this study. Our study also suggested that optimal CA19-9 response and more chemotherapy cycles were associated with favorable outcomes.

The MPACT trial (20) and the PRODIGE trial (21) have reported the effectiveness of AG and FOLFIRINOX regimens in patients with metastatic pancreatic cancer, and those two regimens are category 2A recommendations for LAPC patients according to the 2021 National Comprehensive Cancer Network (NCCN) guidelines (22). As the first prospective study, LAPACT validated AG as an active regimen for treating LAPC and the reported median PFS and OS were 10.9 and 18.8 months, respectively (9). More recently, the NEOLAP trial further demonstrated that induction chemotherapy with AG or AG followed by FOLFIRINOX are both active and safe for patients with LAPC and about a third of patients achieved a surgical conversion (11). In 2021, a randomized trial by Stefano and colleagues reported that nab-paclitaxel/gemcitabine was superior to gemcitabine alone in LAPC patients (23). Although a meta-analysis of FOLFIRINOX in LAPC showed a median OS of 24.2 months which was superior to previous reports (24). Considering the relatively severe toxicities of FOLFIRINOX (25), we preferred AG regimen to be the first-line chemotherapy regimen. Compared to the LAPACT trial, we yielded a longer median PFS of 12.8 months, which might be due to the high local control rate achieved by the chemoradiotherapy. The median OS of 16.3 months in our study seemed to be shorter than LAPACT (16.3 months vs. 18.8 months), but were similar to previous studies, which ranged from 11.1 to 16.5 months (13, 26, 27).

As a method to control local disease, the application of radiotherapy is debatable. A most recent trial, the LAP07 demonstrated an improvement in local control but failed to show survival benefit (13). Previous studies have demonstrated the role of helical tomotherapy in improving disease control efficacy, meanwhile minimizing toxicity (28–30). Additionally, recent studies verified that higher radiation dose resulted in better outcomes (31–33). Based on these reports, we adopted the helical tomotherapy in the current study, which provided highly conformal radiotherapy and relatively high biologically effective dose (BED = 75 Gy) and image guidance. TVI was at high risk of recurrence and one third of patients treated by radiotherapy developed recurrences near the celiac trunk and superior mesenteric artery (SMA) (34). TVI was included in the high dose field in our study and only 1 patient was local recurrence, showing an excellent local disease control (95.5%). The hypofractionated radiotherapy with SIB allowed high dose in tumor volume at high risk of recurrence and meanwhile shortened the treatment duration and enhanced compliance of patients. At the same time, no severe acute or late radiotherapy-related adverse events were observed in our study, which was dependent on the strict dose constrains of duodenum and intestine through treatment planning system of helical tomotherapy. Overall, this retrospective analysis further demonstrated AG plus the hypofractionated tomotherapy was suitable for patients with LAPC.

Furthermore, we analyzed the correlations between characteristics and outcomes of patients *via* univariate and multivariate analyses. A retrospective study including patients treated by gemcitabine-based combinations for borderline or locally advanced pancreatic cancer showed a higher CA19-9 reduction, resulted in a longer survival (35). Similarly, we found most patients experienced a reduction in CA19-9 levels after treatment, and the CA19-9 response was an independent prognostic factor for outcomes of patients in our study. Moreover, we found that more chemotherapy cycles were associated with better survival, which were in line with previous findings (36, 37). Similar findings were also indicated in other types of tumor such as gastric cancer. A phase 3 trial REGATTA compared gastrectomy plus chemotherapy versus chemotherapy for advanced gastric cancer with a single non-curable factor. The results of this trial suggested that the impaired compliance with chemotherapy accounted for the worse survival (38).

Nevertheless, there are several limitations in this study. Firstly, our study was limited by its respective nature and the relatively small sample size. Secondly, in accordance with many retrospective studies, it is hard to capture all side effects because of the incomplete records during treatment and the subsequent recall bias, especially the nonhematological toxicities. In addition, this was a single arm study and lacked comparison with control cohort. Therefore, a prospective study is worthy to be initiated in the future.

In conclusion, our study demonstrated the efficacy and safety of the novel regimen (nab-paclitaxel/gemcitabine plus hypofractionated tomotherapy with SIB) in patients with LAPC, and the promising data inspired us to conduct a prospective trial in the near future.

## Data Availability Statement

The raw data supporting the conclusions of this article will be made available by the authors, without undue reservation.

## Ethics Statement

The studies involving human participants were reviewed and approved by the Ethics Committee of Nanjing Drum Tower Hospital. The patients/participants provided their written informed consent to participate in this study. Written informed consent was obtained from the individual(s) for the publication of any potentially identifiable images or data included in this article.

## Author Contributions

Conceptualization, D.J. and L.B.R. Data curation, S.Z., Q.X., L.C.C., and L.J. Formal analysis, S.Z. and Y.J. Funding acquisition, D.J. Validation, K.W.W. Writing—Original Draft, S.Z. Supervision, D.J. and L.B.R. Writing—Review and Editing, Y.J. and D.J. All authors listed have made a substantial, direct, and intellectual contribution to the work and approved it for publication.

## Funding

This study was supported by the CHEN Xiaoping Foundation for the Development of Science and Techonology of Hubei Province (No. CXPJJH11900001-2019101), the Special Fund of Health Science and Technology Development of Nanjing (No. YKK20080), the National Natural Science Foundation of China (grant number 82072926), and the Natural Science Foundation of Jiangsu Province (grant number BK20191114).

## Conflict of Interest

The authors declare that the research was conducted in the absence of any commercial or financial relationships that could be construed as a potential conflict of interest.

## Publisher’s Note

All claims expressed in this article are solely those of the authors and do not necessarily represent those of their affiliated organizations, or those of the publisher, the editors and the reviewers. Any product that may be evaluated in this article, or claim that may be made by its manufacturer, is not guaranteed or endorsed by the publisher.
