# Supplementary material for: Use of Nab-Paclitaxel Plus Gemcitabine Followed by Hypofractionated Tomotherapy With Simultaneous Integrated Boost in Patients With Locally Advanced Pancreatic Cancer
Source: Front Oncol. 2022 Mar 1;12:782730. doi: 10.3389/fonc.2022.782730 (PMC8922029; doi:10.3389/fonc.2022.782730)
Supplement: Supplementary file 1 [file DataSheet_1.docx]

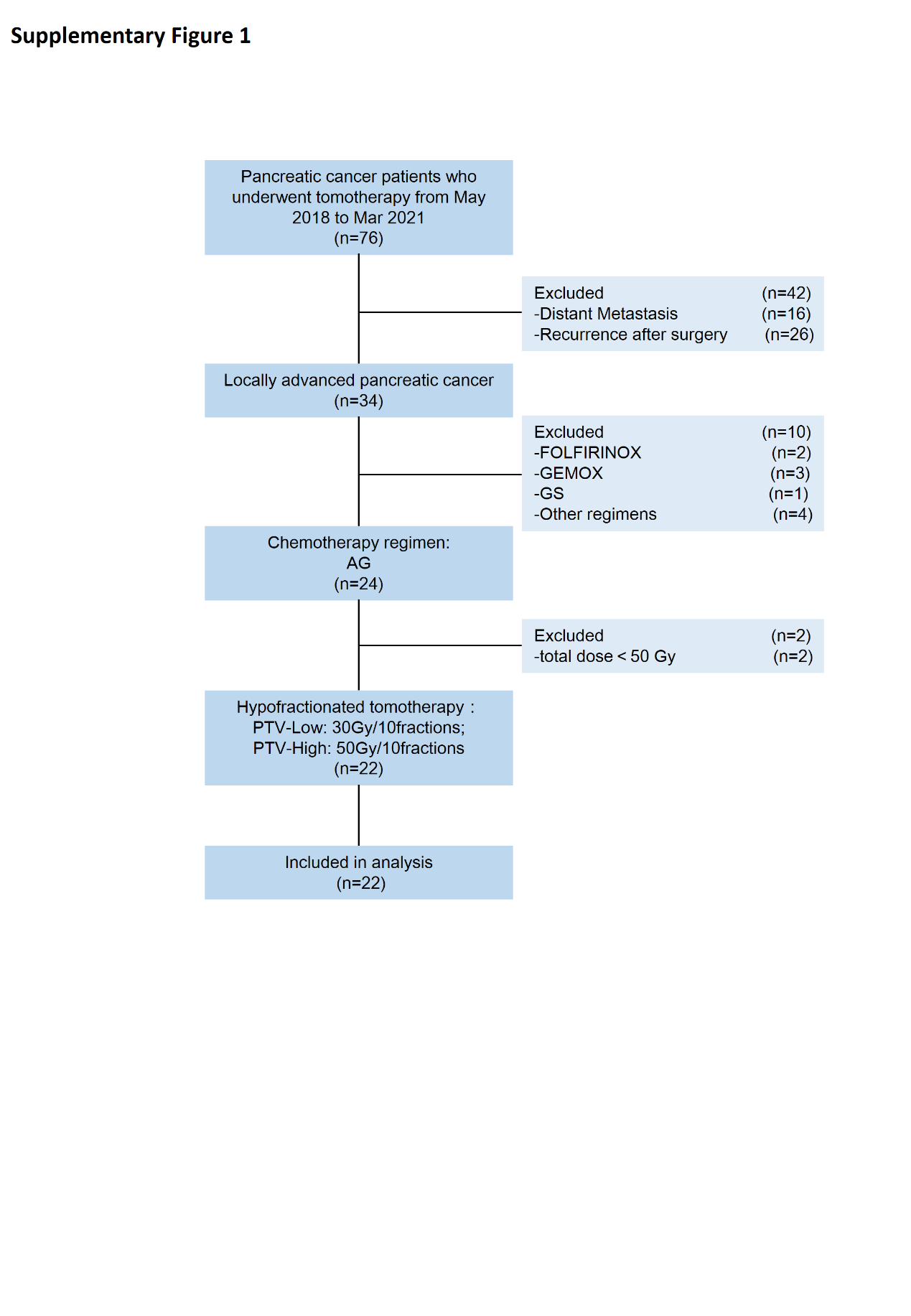


**Supplementary Figure 1. Flowchart of the recruitment process.**

Abbreviations: FOLFIRINOX, fluorouracil plus leucovorin, irinotecan, oxaliplatin; GEMOX, gemcitabine plus oxaliplatin; GS, gemcitabine plus S-1; AG, nab-paclitaxel plus gemcitabine.

Supplentary Table 1. Best response during chemoradiotherapy

|  | **Cases** | **%** |
| --- | --- | --- |
| Rate of objective response | 5 | 22.7 |
| Rate of disease control | 20 | 90.9 |
| Response | | |
| Complete response | 0 | 0 |
| Partial response | 5 | 22.7 |
| Stable disease | 15 | 68.2 |
| Progressive disease | 2 | 9.1 |

Supplementary Table 2. Most common hematological toxicities in 22 patients treated with chemoradiotherapy

| Toxicities | Grade | | | |
| --- | --- | --- | --- | --- |
|  | **0** | **1** | **2** | **≥3** |
|  | **Cases (%)** | **Cases (%)** | **Cases (%)** | **Cases (%)** |
| Hematological | | | | |
| Anemia | 3 (13.6) | 11 (50.0) | 8 (36.4) | 0 (0) |
| Neutropenia | 6 (27.3) | 8 (36.4) | 7 (31.8) | 1 (4.5) |
| Leucopenia | 9 (40.9) | 10 (45.5) | 3 (13.6) | 0(0) |
| Thrombocytopenia | 11 (50.0) | 4 (18.2) | 4 (18.2) | 3 (13.6) |
| Nonhematological | | | | |
| Diarrhea | 21 (95.5) | 1 (4.5) | 0 (0) | 0 (0) |
| Nausea | 16 (72.8) | 3 (13.6) | 3 (13.6) | 0 (0) |
| Vomiting | 15 (68.3) | 4 (18.2) | 2 (9.0) | 1 (4.5) |
| Fatigue | 14 (63.7) | 6 (27.3) | 2 (9.0) | 0 (0) |
| Hepatotoxicity | 16 (72.8) | 4 (18.2) | 2 (9.0) | 0 (0) |
| Peripheral neuropathy | 17 (77.4) | 3 (13.6) | 1 (4.5) | 1 (4.5) |
| Alopecia | 19 (86.5) | 2 (9.0) | 1 (4.5) | 0 (0) |
